# Supplementary material for: Quality Evaluation of Shiitake Blanched and Centrifuged Broths as Functional Instant Drinks
Source: Foods. 2023 Aug 1;12(15):2925. doi: 10.3390/foods12152925 (PMC10418950; doi:10.3390/foods12152925)
Supplement: Supplementary file 1 [file foods-12-02925-s001.zip › foods-2496019-supplementary.pdf]

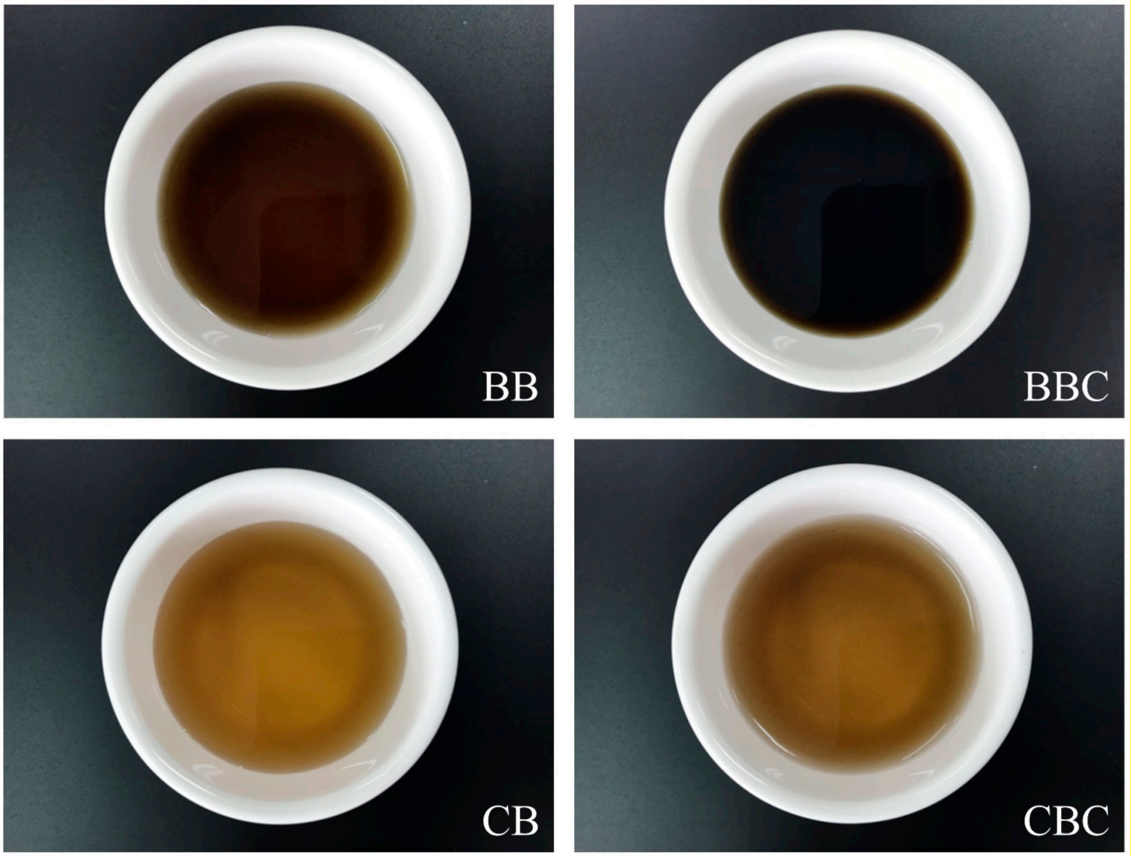

Figure S1. The color of blanched and centrifuged broths and their concentrates.
BB, Blanched broth. BBC, Blanched broth concentrate. CB, Centrifuged broth. CBC, Centrifuged broth concentrate.

Table S1. Moisture, water activity and color property of instant drink powders.

	FS-2[1]	BB-SD13	BB-SD14	BB-SD15	CB-SD13	CB-SD14	CB-SD15
Moisture (%)	1.73 ± 0.03f[2]	7.06 ± 0.05a	5.47 ± 0.01b	4.41 ± 0.05c	3.55 ± 0.06d	2.06 ± 0.05e	1.72 ± 0.01f
Water activity	0.225 ± 0.002e	0.365 ± 0.015a	0.328 ± 0.002b	0.315 ± 0.003c	0.307 ± 0.001c	0.277 ± 0.001d	0.226 ± 0.001e
Color property							
*L**	95.51 ± 0.11a	86.88 ± 0.60g	89.16 ± 0.19f	89.81 ± 0.17e	91.65 ± 0.08d	92.28 ± 0.10c	92.81 ± 0.18b
*a**	-0.61 ± 0.02d	0.44 ± 0.02a	0.14 ± 0.02b	-0.03 ± 0.03c	-0.60 ± 0.02d	-0.61 ± 0.02d	-0.62 ± 0.01d
*b**	3.31 ± 0.03g	10.62 ± 0.15a	8.95 ± 0.11b	8.18 ± 0.13d	8.55 ± 0.14c	7.75 ± 0.02e	7.15 ± 0.04f
*WI**	94.39 ± 0.07a	83.11 ± 0.39g	85.94 ± 0.17f	86.93 ± 0.07e	88.03 ± 0.05d	89.04 ± 0.08c	89.84 ± 0.10b
*ΔE**		11.36 ± 0.38a	8.53 ± 0.16b	7.52 ± 0.06c	6.51 ± 0.07d	5.49 ± 0.07e	4.70 ± 0.07f
Hedonic test[3]		7.4 ± 1.1a	7.6 ± 1.1a	7.4 ± 1.0a	7.4 ± 0.8a	7.6 ± 0.9a	7.6 ± 0.9a

[1]FS-2: Fibersol-2. SD: Spray drying. 13, 14, and 15: dry solids of blanched broth (or centrifuged broth) concentrate/indigestible dextrin (w/w).

[2]Each value is expressed as mean ± standard deviation (n = 3). Values with different lowercase letters within a row differ significantly different ($p < 0.05$).

[3]Hedonic test: each value is expressed as mean ± standard deviation (n = 90). Values with different lowercase letters within a row differ significantly ($p < 0.05$). Nine-point hedonic scale with 1, 5, and 9 representing extremely dislike, neither like nor dislike, and extremely like, respectively.

Table S2. Color property of instant drinks prepared from instant drink powders with hot water.

	*L**	*a**	*b**	*h (°)**	*c**	*ΔE*
Blanched broth						
BB-SD13-1.0[1]	90.84 ± 0.07A[2]	-0.75 ± 0.05F	25.98 ± 0.06F	91.65 ± 0.11A	25.99 ± 0.06F	
BB-SD13-1.5	86.79 ± 0.06B	0.51 ± 0.02E	35.94 ± 0.05E	89.19 ± 0.01B	35.94 ± 0.05E	10.82 ± 0.07E
BB-SD13-2.0	82.80 ± 0.02C	2.45 ± 0.02D	44.55 ± 0.06D	86.85 ± 0.02C	44.62 ± 0.06D	20.48 ± 0.06D
BB-SD13-2.5	79.06 ± 0.03D	4.78 ± 0.02C	51.41 ± 0.06C	84.68 ± 0.02D	51.64 ± 0.06C	28.57 ± 0.07C
BB-SD13-3.0	75.67 ± 0.03E	7.15 ± 0.02B	56.82 ± 0.06B	82.82 ± 0.01E	57.27 ± 0.06B	35.26 ± 0.06B
BB-SD13-3.5	73.14 ± 0.07F	9.15 ± 0.04A	60.34 ± 0.05A	81.38 ± 0.03F	61.03 ± 0.05A	39.89 ± 0.05A
BB-SD14-1.0	90.75 ± 0.04A	-0.80 ± 0.01F	26.39 ± 0.04F	91.73 ± 0.01A	26.40 ± 0.04F	
BB-SD14-1.5	86.78 ± 0.01B	0.48 ± 0.01E	36.13 ± 0.06E	89.24 ± 0.01B	36.13 ± 0.06E	10.59 ± 0.05E
BB-SD14-2.0	83.05 ± 0.03C	2.32 ± 0.04D	44.23 ± 0.14D	86.99 ± 0.04C	44.29 ± 0.14D	19.68 ± 0.14D
BB-SD14-2.5	79.35 ± 0.01D	4.64 ± 0.01C	51.04 ± 0.05C	84.80 ± 0.01D	51.25 ± 0.05C	27.69 ± 0.04C
BB-SD14-3.0	75.87 ± 0.06E	7.07 ± 0.04B	56.49 ± 0.08B	82.87 ± 0.03E	56.93 ± 0.08B	34.49 ± 0.10B
BB-SD14-3.5	72.77 ± 0.05F	9.36 ± 0.05A	60.48 ± 0.08A	81.20 ± 0.04F	61.20 ± 0.08A	39.86 ± 0.10A
BB-SD15-1.0	88.41 ± 0.01A	-0.18 ± 0.01F	33.10 ± 0.03F	90.31 ± 0.01A	33.10 ± 0.03F	
BB-SD15-1.5	86.62 ± 0.36B	0.60 ± 0.15E	37.19 ± 0.86E	89.08 ± 0.21B	37.19 ± 0.86E	4.53 ± 0.94E
BB-SD15-2.0	82.23 ± 0.08C	2.88 ± 0.02D	46.54 ± 0.02D	86.46 ± 0.03C	46.64 ± 0.02D	15.11 ± 0.04D
BB-SD15-2.5	78.41 ± 0.06D	5.35 ± 0.04C	53.43 ± 0.07C	84.28 ± 0.04D	53.69 ± 0.07C	23.32 ± 0.09C
BB-SD15-3.0	74.26 ± 0.01E	8.39 ± 0.02B	59.34 ± 0.04B	81.95 ± 0.02E	59.93 ± 0.04B	31.03 ± 0.03B
BB-SD15-3.5	71.78 ± 0.38F	10.09 ± 0.01A	62.12 ± 0.53A	80.77 ± 0.08F	62.94 ± 0.52A	35.00 ± 0.62A
Centrifuged broth						
CB-SD13-1.0	96.89 ± 0.02A	-2.17 ± 0.01C	14.71 ± 0.02F	98.40 ± 0.02A	14.87 ± 0.02F	
CB-SD13-1.5	95.33 ± 0.02B	-2.60 ± 0.01E	21.06 ± 0.04E	97.03 ± 0.01B	21.22 ± 0.04E	6.55 ± 0.04E
CB-SD13-2.0	93.71 ± 0.01C	-2.80 ± 0.01F	27.32 ± 0.05D	95.85 ± 0.01C	27.46 ± 0.05D	13.02 ± 0.05D
CB-SD13-2.5	92.28 ± 0.01D	-2.56 ± 0.01D	32.30 ± 0.04C	94.54 ± 0.02D	32.40 ± 0.04C	18.19 ± 0.04C
CB-SD13-3.0	90.68 ± 0.02E	-1.98 ± 0.01B	37.27 ± 0.03B	93.04 ± 0.01E	37.32 ± 0.03B	23.40 ± 0.04B
CB-SD13-3.5	88.85 ± 0.03F	-1.20 ± 0.01A	43.23 ± 0.12A	91.59 ± 0.02F	43.25 ± 0.12A	29.65 ± 0.12A
CB-SD14-1.0	96.85 ± 0.06A	-2.23 ± 0.01C	14.94 ± 0.05F	98.50 ± 0.01A	15.10 ± 0.05F	
CB-SD14-1.5	95.19 ± 0.03B	-2.66 ± 0.04E	21.40 ± 0.21E	97.08 ± 0.04B	21.56 ± 0.21E	6.68 ± 0.21E
CB-SD14-2.0	93.63 ± 0.01C	-2.72 ± 0.02F	27.57 ± 0.08D	95.63 ± 0.02C	27.70 ± 0.08D	13.05 ± 0.08D
CB-SD14-2.5	92.07 ± 0.01D	-2.54 ± 0.01D	33.43 ± 0.13C	94.35 ± 0.01D	33.53 ± 0.13C	19.11 ± 0.12C
CB-SD14-3.0	90.41 ± 0.03E	-1.97 ± 0.01B	38.77 ± 0.11B	92.90 ± 0.01E	38.82 ± 0.11B	24.69 ± 0.11B
CB-SD14-3.5	88.81 ± 0.01F	-1.27 ± 0.03A	43.99 ± 0.24A	91.66 ± 0.03F	44.01 ± 0.24A	30.16 ± 0.23A
CB-SD15-1.0	96.55 ± 0.01A	-2.26 ± 0.01C	15.79 ± 0.02F	98.16 ± 0.02A	15.95 ± 0.02F	

CB-SD15-1.5	95.05 ± 0.36B	-2.68 ± 0.06DE	22.25 ± 1.40E	96.88 ± 0.29B	22.41 ± 1.39E	6.65 ± 1.44E
CB-SD15-2.0	93.62 ± 0.02C	-2.79 ± 0.01E	27.86 ± 0.06D	95.71 ± 0.01C	28.00 ± 0.06D	12.44 ± 0.06D
CB-SD15-2.5	92.00 ± 0.02D	-2.57 ± 0.01D	34.00 ± 0.13C	94.33 ± 0.01D	34.10 ± 0.13C	18.78 ± 0.13C
CB-SD15-3.0	90.11 ± 0.25E	-1.96 ± 0.10B	40.20 ± 0.70B	92.80 ± 0.19E	40.25 ± 0.69B	25.25 ± 0.74B
CB-SD15-3.5	89.13 ± 0.44F	-1.55 ± 0.22A	43.83 ± 1.36A	92.03 ± 0.36F	43.86 ± 1.35A	29.02 ± 1.43A

[1]SD: Spray drying. 13, 14, and 15: dry solid of blanched broth (or centrifuged broth) concentrate/indigestible dextrin (w/w). 1.0, 1.5, 2.0, 2.5, 3.0, and 3.5: instant drinks containing 1.0%, 1.5%, 2.0%, 2.5%, 3.0%, and 3.5% dried broth, respectively.

[2]Each value is expressed as mean ± standard deviation (n = 3). Values with different capital letters within a column differ significantly different ($p < 0.05$).
